# Update on the Research of an Emulgel for the Effective Treatment of Atopic Dermatitis: Clinical Investigation in Children

**DOI:** 10.3390/gels11110880

**Published:** 2025-11-02

**Authors:** Almudena Gómez-Farto, Ana Leticia Jiménez-Escobar, Noelia Pérez-González, Amy Lozano-White, Jésica Expósito-Herrera, Trinidad Montero-Vílchez, Beatriz Clares, Salvador Arias-Santiago

**Affiliations:** 1 Institute for Biotechnology, Pharmaceutical and Orphan Drug Research, S.L., 18016 Granada, Spain; 2 Department of Pharmacy & Pharmaceutical Technology, Faculty of Pharmacy, University of Granada, 18071 Granada, Spain; 3 Health Centre La Chana, 18015 Granada, Spain; 4 Health Centre Cijuela, 18339 Granada, Spain; 5 Biosanitary Institute of Granada, 18012 Granada, Spainsalvadorarias@ugr.es (S.A.-S.); 6 Department of Dermatology, Granada School of Medicine, University of Granada, Virgen de las Nieves University Hospital, 18016 Granada, Spain; 7 Institute of Nanoscience and Nanotechnology (IN2UB), University of Barcelona, 08028 Barcelona, Spain

**Keywords:** atopic dermatitis, emulgel, skin barrier function, children, epidermal growth factor, hyaluronic acid, eczema

## Abstract

Atopic dermatitis (AD) is a chronic inflammatory skin condition that affects up to 25% of children and impairs both skin barrier function and quality of life. This study examined the effectiveness of an emulgel containing hyaluronic acid, glycerol, grape seed oil, *Calendula officinalis*, aloe vera and sh-oligopeptide-1 (a synthetic Epidermal Growth Factor) for treating paediatric AD. In a randomised, self-controlled trial, 57 children (aged 2–14) applied the emulgel twice daily for 10 days to one forearm and left the other forearm as a control. Skin barrier parameters such as transepidermal water loss (TEWL), stratum corneum hydration (SCH), erythema and pH were measured. After applying the emulgel, lesional skin showed reduced erythema (*p* = 0.007), lower TEWL (*p* = 0.002) and higher SCH (*p* < 0.001). Non-lesional skin showed improved SCH (*p* < 0.001). SCORing Atopic Dermatitis (SCORAD) and Eczema Area and Severity Index (EASI) scores indicated milder disease post-treatment (mild cases: 64.9% to 80.7% SCORAD; 82.5% to 93.0%EASI). The Dermatology Life Quality Index improved by ~3.5 points, and patients reported high satisfaction with no adverse effects. This emulgel is an effective and well-tolerated adjunctive therapy for paediatric AD, enhancing barrier function and clinical outcomes.

## 1. Introduction

Atopic dermatitis (AD), also known as atopic eczema, is a chronic inflammatory skin condition characterised by impaired skin barrier function, intense itchiness and eczematous lesions. It is associated with an increased risk of comorbid allergic conditions, including asthma, rhinitis and food allergies in children [1,2]. The prevalence of AD varies by geographic region, affecting up to 25% of children and 10% of adults [3]. Approximately 85% of AD cases manifest before the age of five, with about 25% of patients experiencing symptoms into adulthood [3,4].

While core features of AD, such as flexural eczema, comorbid atopy, and xerosis, are similar in children and adults, notable differences exist between the two populations. These differences partly stem from genetic and immune system dysregulation. Adults typically exhibit more chronic disease manifestations, hand eczema, and a stronger correlation between disease activity and emotional factors. In contrast, children often present with exudative lesions, perifollicular accentuation, pityriasis alba, Dennie–Morgan folds, and a seborrheic dermatitis-like presentation [4]. In adult-onset AD, the most affected body regions include the hands, eyelids, neck, and inner upper arms, whereas childhood-onset AD tends to be less specific to these areas, primarily affecting flexural regions [5,6,7].

Approximately 50% of children with AD report that their condition negatively impacts their quality of life [8]. Moreover, the management of paediatric AD is often limited by the severity of side effects associated with current therapeutic options [9,10].

AD is characterised by increased transepidermal water loss (TEWL), erythema and reduced stratum corneum hydration (SCH) due to skin barrier dysfunction [11,12]. One of the five pillars of AD management, according to the Asia Pacific Consensus, is the rebuilding and maintenance of optimal barrier function [13].

A previous study demonstrated that an emulgel containing hyaluronic acid, glycerol, grape seed oil, *Calendula officinalis*, aloe vera and sh-oligopeptide-1 improved skin condition in adults with AD [14]. This improvement was evidenced by changes in homeostatic parameters, including skin temperature, TEWL and SCH, as well as reductions in SCORAD and EASI scores after 10 days of treatment. However, a limitation of this study was the lack of data on the emulgel’s efficacy in children.

This study aims to evaluate the efficacy of the same emulgel in treating paediatric AD, building on its established effectiveness in adults.

## 2. Results and Discussion

**Figure 1:**
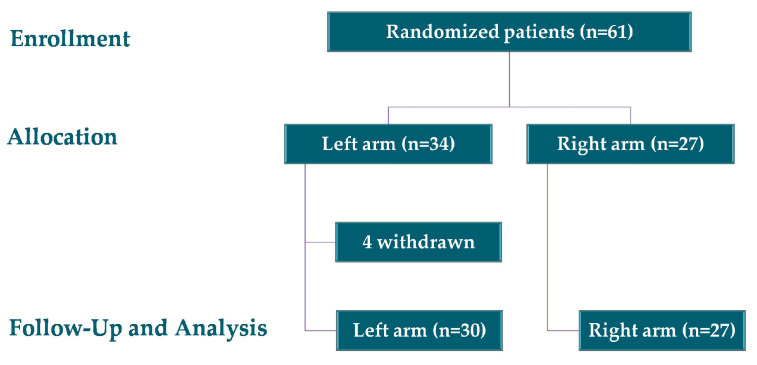
Overview of participant inclusion, group assignment, and final analysis.

Between January 2023 and July 2025, 61 children were enrolled in the study and randomised into two groups: 34 in the left arm group and 27 in the right arm group. Four participants withdrew voluntarily, resulting in a final sample of 57 children, 30 in the left arm group and 27 in the right arm group, as illustrated in Figure 1.

The mean age of participants was 6.72 ± 3.82 years. As shown in Table 1, 59.6% of participants were female (*n* = 34) and 40.4% were male (*n* = 23). Regarding skin phototype, most children were classified as type II (43.9%) or III (33.3%), with phototypes I, IV, V, and VI being less common (17.5%, 1.8%, 1.8%, and 1.8%, respectively).

Sun exposure was reported as frequent in 24.6% of cases, occasional in 68.4%, and absent in 7.0%. Regarding moisturiser use, 50.9% of children applied it once daily, 22.8% applied it multiple times daily, 15.8% used it occasionally, and 5.3% rarely or never used it.

At baseline, 43.9% of participants were not receiving any treatment, while 43.9% were using topical therapy, primarily corticosteroids. Additionally, 5.3% were treated with cyclosporine, and 7.0% received biological therapy.

The most common comorbidities were allergies (29.8%) and asthma (22.8%), followed by contact dermatitis (7%), rhinitis (3.5%), and conjunctivitis (3.5%). Only one child presented with diaper dermatitis, and no cases of prurigo nodularis were reported.

Table 2 summarises the changes in skin barrier homeostasis parameters before and after the application of the emulgel, covering both non-lesional and lesional zones presenting eczema.

**Figure 2:**
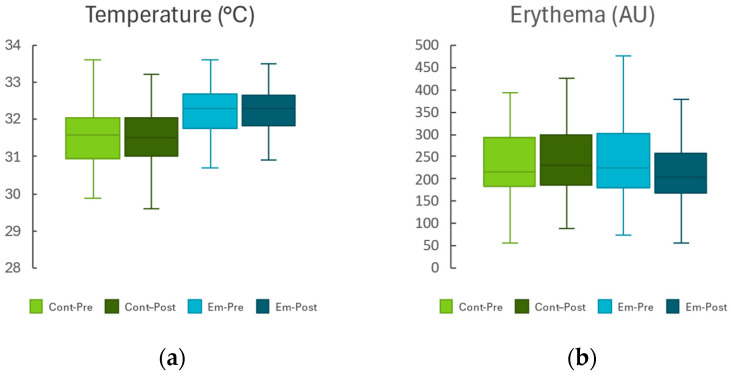

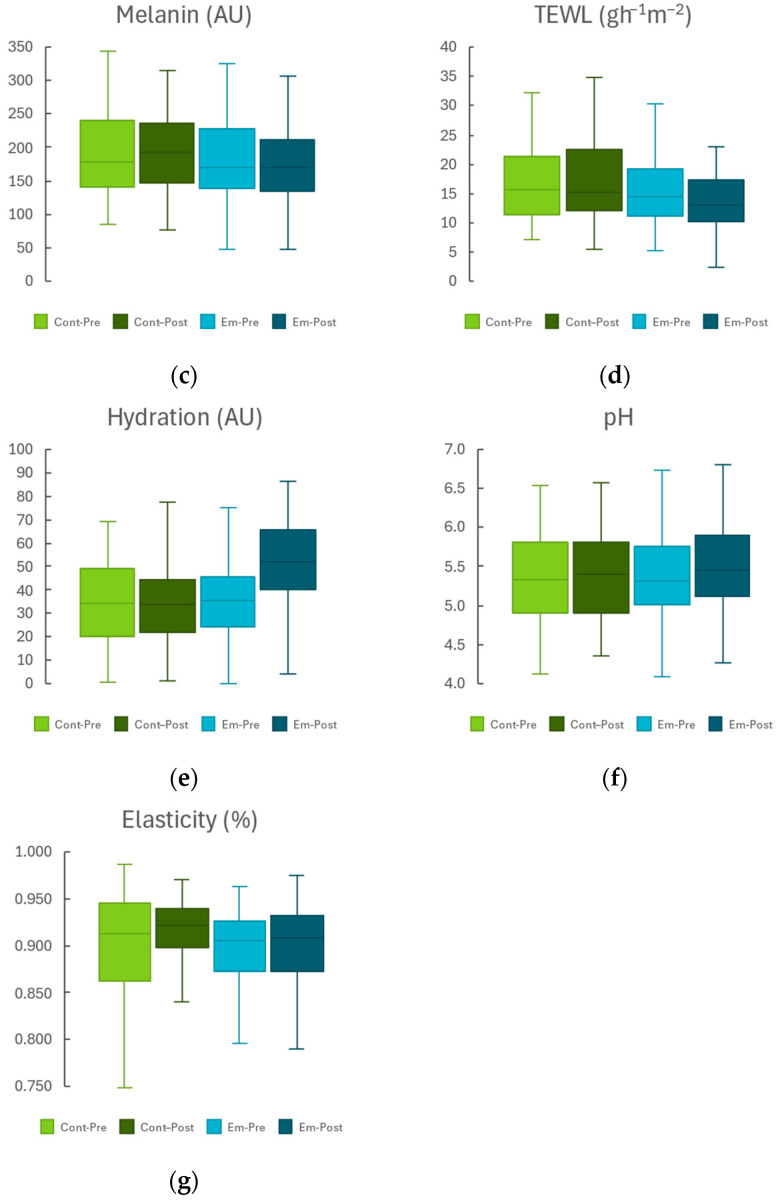
Changes in skin homeostasis parameters in eczematous areas before and after treatment: (**a**) Skin temperature (°C), (**b**) Erythema (arbitrary units, AU), (**c**) Melanin content (AU), (**d**) Transepidermal water loss (TEWL, g·h^−1^·m^−2^), (**e**) Stratum corneum hydration (AU), (**f**) Skin surface pH, and (**g**) Skin elasticity (%). The boxplots show the values at baseline (Pre) and after 10 days (Post) of twice-daily application to the control (Cont) and treated (Em) areas. Light/dark green indicates Cont-Pre/Cont-Post, and light/dark blue Em-Pre/Em-Post. Data are expressed as mean ± SD.

Figure 2 shows a bar chart comparing each homeostasis parameter between the intervention and control groups before and after emulgel application.

**Figure 3:**
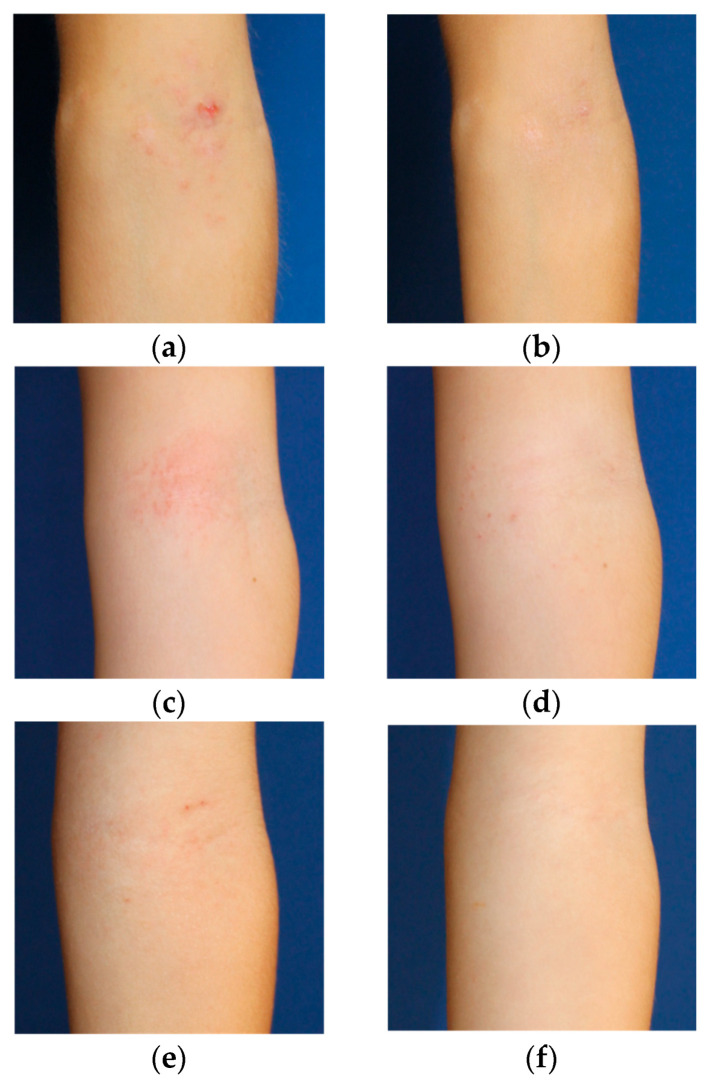
Clinical images illustrating the improvement in appearance and erythema of the lesional areas. (**a**) Forearm patient 1 before treatment; (**b**) Forearm patient 1 after treatment; (**c**) Forearm patient 2 before treatment; (**d**) Forearm patient 2 after treatment; (**e**) Forearm patient 3 before treatment; (**f**) Forearm patient 3 after treatment.

Figure 3 shows photographs of patients taken before and after treatment. These images illustrate the clinical improvement observed when using the study formulation, showing a visible reduction in erythema and lesion severity. The examples demonstrate the overall improvement in skin condition achieved during the treatment period.

### 2.1. Non-Lesional Skin Area

Most parameters showed no significant changes in either the emulgel-treated or untreated (control) forearm. However, emulgel treatment resulted in a highly significant increase in SCH (Δ: 10.13 ± 1.31 AU; *p* < 0.001), indicating enhanced skin moisture retention. This improvement was statistically significant compared to the control forearm (*p* < 0.001). A slight reduction in TEWL was observed but did not reach statistical significance, suggesting a trend toward improved skin barrier integrity.

The significant increase in SCH confirms the moisturising efficacy of the emulgel, attributable to humectants such as hyaluronic acid, a key component of the extracellular matrix that maintains skin hydration [15] and glycerol, a natural moisturising factor that enhances lipid cohesion in the stratum corneum [16]. The lack of significant changes in other parameters (e.g., TEWL, erythema, pH and elasticity) in non-lesional skin may reflect a relatively balanced homeostatic system, with limited scope for improvement beyond hydration.

### 2.2. Lesional Skin Area

In eczema-affected skin, emulgel treatment yielded significant improvements. Erythema decreased markedly (Δ: −26.48 ± 9.38 AU; *p* = 0.007), TEWL was significantly reduced (Δ: −5.59 ± 1.69 g·h^−1^·m^−2^; *p* = 0.002), and SCH increased substantially (Δ: 17.44 ± 2.14 AU; *p* < 0.001). These changes were statistically significant compared to the control arm. Additionally, a slight increase in skin pH was observed in the emulgel-treated arm (Δ: 0.10 ± 0.07; *p* < 0.020). These findings suggest that the barrier function is being restored and that inflammation in AD lesions is decreasing.

These findings indicate that the emulgel exerts both anti-inflammatory and barrier-restorative effects, which are critical in AD, where skin barrier dysfunction and inflammation are mutually reinforcing. This is to be expected given that emollient-based therapy remains a cornerstone of AD management [13].

The emulgel’s efficacy is likely due to its combination of active components. Glycerol enhances skin hydration, reduces TEWL, and improves biomechanical properties [17]. Hyaluronic acid plays a key role in maintaining the normal structure of the stratum corneum and the epidermal barrier function [18]. Polyphenols, with their antioxidant, anti-inflammatory, and antibacterial properties, contribute to these effects [19]. *Calendula officinalis* possesses anti-inflammatory, anti-irritant and skin regeneration properties [20,21], while *Aloe vera* is used to treat eczemas due to its anti-inflammatory, antimicrobial, and wound healing properties [22]. Additionally, sh-oligopeptide-1, a recombinant human peptide, whose starting gene is identical to the human gene that codes for Epidermal Growth Factor (EGF), contributes to these effects because EGF has been shown in vitro to reduce TEWL, epidermal thickness, AD-related inflammation, and total and allergen-specific immunoglobulin E [23].

In AD patients, skin pH often shifts toward neutral to basic ranges. Maintaining an acidic pH (4.0–5.8) is critical for preserving stratum corneum integrity, barrier function, and microbial balance [24,25]. In this study, the baseline skin pH was approximately 5.4 across all subjects, within the optimal range, and no significant changes were observed post-treatment. The slight pH increase in lesional skin (Δ: 0.10 ± 0.07; *p* < 0.020) is not considered clinically significant because skin pH remained within the acidic range.

This paediatric study demonstrates that emulgel treatment significantly enhances skin barrier homeostasis in children with AD, particularly in lesional skin. The emulgel, based on hyaluronic acid, glycerol, *Calendula officinalis*, *Aloe vera*, polyphenols and sh-oligopeptide-1, significantly improved TEWL, SCH and erythema in lesional skin. These findings are consistent with Gómez-Farto et al., who reported statistically significant improvements in TEWL (*p* = 0.006), erythema (*p* = 0.008) and hydration (*p* < 0.001) using the same formulation in older AD patients (>14 years) [14]. Regular use of emollient formulations in mild to moderate AD has been shown to reduce TEWL, enhance skin hydration, prolong the period between relapses, alleviate symptom severity, and reduce flare-ups and corticosteroid use [26,27].

**Figure 4:**
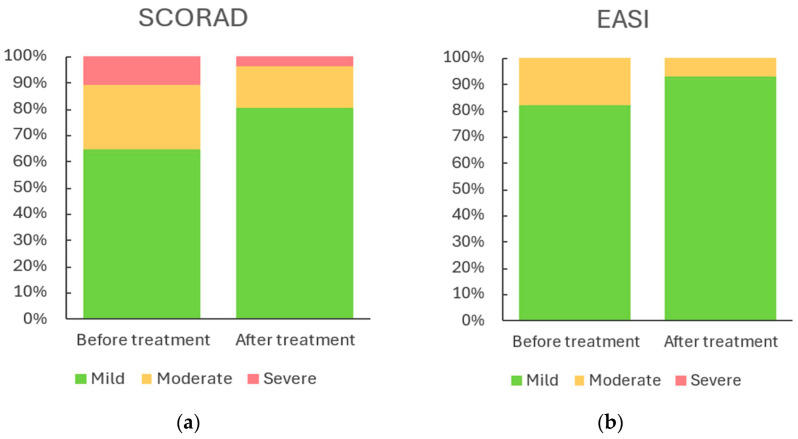
Distribution of disease severity at baseline and after treatment (*n* = 57). (**a**) Based on SCORAD; (**b**) Based on EASI. Categories for SCORAD and EASI include Mild, Moderate, and Severe; no Severe cases were observed for EASI.

Table 3 and Figure 4 summarise disease severity according to the SCORAD and EASI before and after treatment. Based on the SCORAD index, the proportion of patients with mild disease increased from 64.9% at baseline to 80.7% after treatment. Conversely, the proportion of patients with moderate disease decreased from 24.6% to 15.8%, while the proportion with severe disease fell from 10.5% to 3.5%.

Similarly, according to the EASI score, the majority of patients were categorised as having mild disease both before and after treatment (82.5% and 93.0%, respectively). The proportion of patients with moderate disease declined from 17.5% to 7.0%, and no patients were classified as having severe disease at either time point.

In this paediatric study, emulgel treatment led to a clinically relevant reduction in disease burden. The proportion of severe cases, as measured by SCORAD index, decreased from 10.5% to 3.5%, while mild cases increased significantly (SCORAD: 64.9% → 80.7%; EASI: 82.5% → 93.0%). These clinical improvements coincided with objective parameters in lesional skin, including reduced TEWL, increased SCH, and decreased erythema, suggesting a treatment effect beyond symptomatic relief. Randomised trials of emollient formulations have similarly demonstrated reductions in clinical indices and corticosteroid use, reinforcing the clinical significance of barrier-directed topical therapies [28,29].

The mean Dermatology Life Quality Index (DLQI) score decreased from 7.3 ± 6.2 at baseline to 3.8 ± 4.2 post-treatment, reflecting an approximate reduction of 3.5 points. The median DLQI score also declined from 6.0 to 3.0, indicating an overall improvement in quality of life. Most participants had lower DLQI scores at the end of the study, suggesting positive impacts on daily activities and emotional well-being. Given that higher DLQI scores indicate greater impairment, this reduction underscores the clinical benefit of the intervention, likely driven by improvements in disease severity and skin condition.

Published studies suggest a minimal clinically important difference (MCID) for DLQI in inflammatory dermatological conditions of approximately 3–4 points, with 4 often considered a conservative threshold [30]. The observed mean reduction of 3.5 points in this paediatric cohort approaches but does not fully meet this threshold. Nevertheless, the consistent improvement in median scores across most participants supports a clinically relevant trend, aligning with objective and clinician-rated outcomes. AD significantly impairs quality of life and psychological well-being in affected children [31,32], often causing sleep disturbances [33] and impacting family members’ quality of life [34]. Thus, these improvements are meaningful for both children and their parents or caregivers.

**Figure 5:**
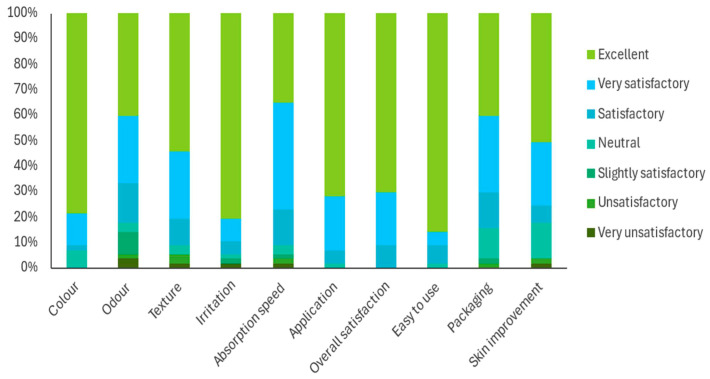
User perception of the product based on multiple evaluated characteristics (*n* = 57).

Figure 5 summarises participants’ evaluations of the formulation. The product received highly positive ratings across most attributes. Notably, 78.9% of participants rated the colour as excellent, and 54.4% rated the texture as excellent. Absorption speed and ease of application were also favourably evaluated, with 42.1% and 71.9% of participants rating these attributes as excellent, respectively. Regarding perceived skin improvement, 50.9% of participants reported excellent outcomes, and 24.6% rated it as ‘very satisfactory’. Reports of irritation were minimal, with 80.7% rating this attribute as excellent. Only a small proportion of participants selected neutral or slightly satisfactory options, and none rated the colour as unsatisfactory. The overall positive perception of the emulgel’s sensory attributes and ease of use likely contributed to participant satisfaction and willingness to use it consistently. These characteristics are critical for enhancing treatment adherence in chronic skin conditions requiring long-term topical therapy. No adverse effects, relapses, or flares were reported.

Patient perception and product acceptability were highly positive, with high ratings for colour, texture and absorption speed, and minimal reports of irritation. Sensory acceptability is a critical determinant of adherence to topical regimens, as poor organoleptic properties are consistently associated with reduced persistence and suboptimal therapeutic outcomes [35,36]. The favourable sensory profile of the emulgel likely contributed to treatment adherence, supporting the observed clinical benefits.

This paediatric study demonstrates consistent improvements in objective barrier parameters, including a significant increase in SCH, a reduction in TEWL, and decreased erythema in lesional skin. These changes coincide with clinically significant reductions in disease severity, SCORAD and EASI values, and improved patient-reported outcomes, DLQI scores and product acceptability. These multimodal improvements support the hypothesis that targeted topical barrier reinforcement translates into meaningful clinical benefits for children with AD.

Barrier dysfunction is a central pathogenic mechanism in AD, linking increased TEWL, reduced hydration, altered pH, and inflammation. Restoring barrier homeostasis offers a biologically plausible approach to reducing disease activity [37]. The emulgel’s ability to enhance SCH, reduce TEWL, and decrease erythema in lesional skin aligns with prior studies showing that well-formulated emollients improve epidermal hydration and reduce objective severity indices. Randomised controlled trials have similarly reported improvements in SCORAD with regular emollient application, with mechanistic studies demonstrating short-term increases in hydration and variable effects on TEWL depending on formulation [38].

These findings collectively support the efficacy of the emulgel in enhancing skin barrier homeostasis and mitigating key pathophysiological features of AD in children. They also highlight its potential as an effective adjunctive therapy for paediatric AD management. The correlation between biophysical improvements and patient-reported symptom relief further reinforces the clinical relevance of these findings.

A key strength of this study is the objective measurement of multiple skin barrier parameters (TEWL, SCH, erythema, pH and elasticity) in both lesional and non-lesional skin before and after treatment, compared against control (untreated) forearms. The paediatric focus is particularly valuable, as children’s skin exhibits developmental differences, including a thinner stratum corneum and epidermis, lower concentration of natural moisturising factors (NMFs) and surface lipids, and higher cell proliferation, making barrier function more vulnerable [39]. Douladiris et al. emphasise that children with AD have higher TEWL and lower skin hydration in both lesional and non-lesional areas compared to healthy skin [40].

Limitations include the moderate sample size (*n* = 57 analysed) and the relatively short follow-up period. While the forearm treated-versus-control design is informative for biophysical measures, it does not substitute for a fully randomised, double-blind, placebo-controlled trial with extended follow-up. Additionally, patients continued their usual AD treatments (topical or systemic), which may confound the emulgel’s specific effects. Finally, although the DLQI improved substantially for many participants, the mean reduction of 3.5 points falls slightly below the conservative MCID of 4 points recommended in the DLQI literature for inflammatory dermatological conditions [30], so the results must be interpreted cautiously.

The concordance of objective outcomes (TEWL, SCH and erythema), clinician-rated outcomes (SCORAD/EASI) and patient-reported outcomes (DLQI and acceptability) suggests that the emulgel is a promising adjunctive and maintenance therapy for paediatric AD. Further study steps should include the following: (1) a longer follow-up period to assess durability and flare prevention; (2) blinded assessment of clinical endpoints (SCORAD/EASI); and (3) quantification of rescue medication use and corticosteroid-sparing effects to further validate the emulgel’s therapeutic impact.

## 3. Conclusions

The findings of this paediatric study demonstrate that the tested emulgel has a consistent barrier-restorative effect. This is reflected in significant improvements in SCORAD, reductions in TEWL and erythema, and partial normalisation of skin pH in affected areas. These objective changes were accompanied by clinically relevant shifts towards milder disease severity, as evidenced by improvements in SCORAD and EASI scores. They were also associated with meaningful gains in patient-reported outcomes, including a reduction in DLQI, and high acceptability of the formulation. Overall, these results support the use of the emulgel as an effective, well-tolerated, adjunctive therapy for paediatric AD, capable of addressing both the pathophysiological features and the quality-of-life dimensions of the condition.

## 4. Materials and Methods

### 4.1. Emulgel Characteristics

The formulation included the following active ingredients at the specified concentrations: hyaluronic acid (1.0%), glycerol (5.0%), polyphenols (0.00072%), *Calendula officinalis* (1.8%), *Aloe vera* (1.0%) and sh-oligopeptide-1 (0.00001%). Various excipients were also included, such as emollients, humectants, surfactants, antioxidants and buffering components. These were dispersed in both the oil and aqueous phases of the emulsion, along with a gelling agent to form the emulgel.

The formulation was an easy-to-apply and absorb light green emulgel, with a pH between 5.5 and 6.5. It had the microbiological properties required for topical products according to ISO 17516:2014 [41].

### 4.2. Study Design

This was a single-centre, randomised, self-controlled clinical trial conducted at the Dermatology Department of Hospital Universitario Virgen de las Nieves (Granada, Spain). The protocol was adapted from one that had previously been validated for adult patients [14] for use with paediatric patients.

Inclusion criteria:

- Children aged 2 to 14 years diagnosed with AD by a dermatologist according to Hanifin and Rajka criteria, with a disease duration of at least six months.
- Patients who had attended the AD clinic at the Dermatology Department of Hospital Universitario Virgen de las Nieves.

Exclusion criteria:

- Presence of any clinical infection in the area designated for measurement.
- History of cancer or immunosuppression.
- Coexisting inflammatory skin conditions such as psoriasis or hidradenitis suppurativa.

Participants who met these criteria and voluntarily agreed to participate were randomly assigned to receive the intervention on either the left or right arm. Randomisation was performed before enrolment using a random number table generated in Microsoft Excel^®^. Even numbers corresponded to allocation of the intervention to the right arm and the control to the left, whereas odd numbers indicated the opposite.

All patients continued their usual treatment for AD, which could include topical or systemic corticosteroids, monoclonal antibodies, or antibiotics as prescribed by their physician. In the experimental arm, the test emulgel was applied twice daily for 10 days in addition to conventional therapy. The control arm received the standard treatment alone, without using any moisturising or repair creams.

At the baseline visit, parents or legal guardians were instructed to apply the emulgel to the designated area on the upper arm indicated by the investigator, avoiding moisturisers on the opposite arm. To standardise skincare practices, participants were advised not to swim in chlorinated pools, take bleach baths, or use topical antibiotic creams during the 7 days prior to study visits, and to refrain from applying moisturisers or bathing/showering within 24 h before each visit.

Assessments were performed at baseline and after 10 days to compare the effects of the intervention on the treated and untreated arms.

### 4.3. Sociodemographic Variables

Sociodemographic data collected included sex, age, and skin phototype. Information regarding skincare habits, such as the frequency of moisturiser use and sun exposure, was also recorded. Personal medical history was also documented, including conditions such as asthma, allergies, nodular prurigo, rhinitis, conjunctivitis, and both contact and diaper dermatitis. Regarding the condition under study, the current treatment for AD was also recorded.

### 4.4. Skin Barrier Function

Epidermal barrier function was assessed through a series of non-invasive bio-physical measurements using probes connected to a multiprobe adapter system (MPA COURAGE+KHAZAKA Electronic GmbH, MICROCAYA S.L., Bilbao, Spain), as described in our previous study [14]. Measurements were taken both on a flexural eczematous lesion located on the volar forearm and on an adjacent, non-lesional skin area.

The recorded parameters included:

- Skin temperature, which reflects changes in microcirculation and tends to rise in cases of barrier dysfunction [42].
- Melanin and erythema index, which are associated with skin exposure to irritants, allergens, or UV radiation [43].
- Transepidermal Water Loss (TEWL), which is an indicator of water diffusion through the stratum corneum, with increased values reflecting impaired barrier function [44].
- Stratum Corneum Hydration (SCH), which is a marker of epidermal water content and related to the lipid organisation in the barrier [44,45].
- Skin surface pH, which is important for lipid organisation and metabolism within the stratum corneum [14].
- Skin elasticity, which reflects the condition of dermal elastic fibres [46].

### 4.5. Severity and Quality of Life

The SCORAD index was used to assess severity, integrating affected area, intensity of clinical signs and subjective symptoms. In a paediatric context, this is classified as mild (0–25), moderate (25–50) or severe (>50).

In addition, the EASI was used to assess the extent and severity of eczema across four anatomical regions: the head and neck, the trunk, the upper limbs, and the lower limbs. The severity categories were defined as follows: mild (<7), moderate (7.1–21), severe (21.1–50) and very severe (>50).

The Dermatology Life Quality Index (DLQI) adapted for children was used in the study. The DLQI is a widely used, validated instrument in clinical practice and trials, and was also administered to evaluate the impact of AD symptoms and treatments on patients’ quality of life. Consisting of 10 questions with four response options (not at all, a little, a lot, very much), it is scored from 0 to 3; the response ‘not relevant’ is scored as 0. The final score is obtained by summing all items (range 0–30), with higher values reflecting greater impairment in quality of life. For interpretation purposes, the total score can also be expressed as a percentage of the maximum possible score (30 points).

### 4.6. User Perception Assessment

Participants evaluated the product by completing a 10-item questionnaire covering different aspects of the formulation, including colour, odour, texture, potential irritation, absorption speed, ease of application, overall satisfaction, ease of use, packaging, and perceived improvement of the skin. Each item was rated on a seven-point scale ranging from very unsatisfactory to excellent. The mean score for each question was calculated and expressed as a percentage. All subjects were asked to answer the questionnaire, which was used as a self-evaluation tool.

### 4.7. Statistical Analyses

Statistical analyses were conducted using IBM SPSS Statistics software v. 26.0 (IBM Corp., Armonk, NY, USA). All study variables were entered into a database for further analysis. The Kolmogorov–Smirnov and Shapiro–Wilk tests were used to examine the distribution of the data and assess normality. Qualitative variables are expressed as absolute and relative frequencies, while quantitative variables are reported as the mean ± standard deviation. Differences in categorical variables were evaluated using the chi-square test. For continuous variables, Student’s *t*-tests were applied: the independent samples *t*-test for comparisons between groups, and the paired *t*-test for within-subject analyses. A two-tailed *p*-value of less than 0.05 was considered statistically significant.

### 4.8. Ethical Analysis

The study complied with the principles of the Declaration of Helsinki and ISO 14155:2020 [47] and received approval from the Andalusian Biomedical Research Ethics Portal (Project identification code 2327-M1-21) and from the Spanish Agency for Medicines and Medical Devices (AEMPS).

As all participants were under 18 years of age, written informed consent was obtained from their parents or legal guardians after they were informed about the study procedures. Only non-invasive measurements were performed, and personal data were handled confidentially at all times.

## Figures and Tables

**Table 1:** Sociodemographic and clinical characteristics of patients. Continuous variables are expressed as the mean ± standard deviation (SD), and nominal variables are expressed as absolute frequencies (relative frequencies).

Characteristics	Mean/No. (*n* = 57)	%
Age (years)	6.72 ± 3.82	
Sex		
Women	34	59.6
Men	23	40.4
Phototype		
I	10	17.5
II	25	43.9
III	19	33.3
IV	1	1.8
V	1	1.8
VI	1	1.8
Sun exposure		
Frequently	14	24.6
Occasionally	39	68.4
Never	4	7.0
Frequency of moisturiser use		
Several times a day	13	22.8
Rarely	3	5.3
Once a day	29	50.9
Sometimes	9	15.8
Never	3	5.3
Treatment		
None	25	43.9
Topical	25	43.9
Cyclosporine	3	5.3
Biological	4	7.0
Comorbidities		
Asthma	13	22.8
Allergies	17	29.8
Prurigo nodularis	0	0
Rhinitis	2	3.5
Conjunctivitis	2	3.5
Contact dermatitis	4	7.0
Diaper dermatitis	1	1.8

**Table 2:** Changes in skin barrier homeostasis parameters before and after treatment in non-lesional and lesional areas of the forearm (*n* = 57).

**Homeostasis** **Parameter**	**Non-Lesional Skin**				
	**Control Forearm**		**Emulgel-Treated Forearm**		*p* *
	Δ*Before*/*After*	*p* ^1^	Δ*Before*/*After*	*p* ^2^	
Temperature (°C)	−0.14 ± 0.14	0.298	−0.19 ± 0.14	0.193	0.693
Erythema (AU)	−0.19 ± 5.24	0.971	−0.66 ± 5.42	0.903	0.941
TEWL (g·m^−2^·h^−1^)	−1.21 ± 0.78	0.128	−1.64 ± 0.83	0.053	0.412
SCH (AU)	−2.18 ± 1.53	0.159	10.13 ± 1.31	**<0.001**	**<0.001**
pH	−0.02 ± 0.09	0.839	−0.03 ± 0.08	0.722	0.820
Elasticity (%)	−0.001 ± 0.008	0.883	−0.006 ± 0.006	0.300	0.587
**Homeostasis** **Parameter**	**Lesional Skin**				
	**Control Forearm**		**Emulgel-Treated Forearm**		*p* *
	Δ*Before*/*After*	*p* ^1^	Δ*Before*/*After*	*p* ^2^	
Temperature (°C)	−0.02 ± 0.13	0.857	−0.05 ± 0.12	0.667	0.781
Erythema (AU)	5.43 ± 8.86	0.542	−26.48 ± 9.38	**0.007**	**0.002**
TEWL (g·m^−2^·h^−1^)	−1.99 ± 0.97	**0.045**	−5.59 ± 1.69	**0.002**	**0.021**
SCH (AU)	0.90 ± 2.06	0.665	17.44 ± 2.14	**<0.001**	**<0.001**
pH	−0.01 ± 0.08	0.930	0.10 ± 0.07	0.151	**0.020**
Elasticity (%)	0.006 ± 0.009	0.497	−0.004 ± 0.008	0.562	0.295

AU, arbitrary units; SCH, Stratum Corneum Hydration; TEWL, Transepidermal Water Loss. ^1^ *p*-value from Student’s paired *t*-test comparing baseline and end-of-follow-up epidermal barrier function parameters in the control forearm. ^2^ *p*-value from Student’s paired *t*-test comparing baseline and end-of-treatment parameters in the emulgel arm. * *p*-value from Student’s paired *t*-test comparing the changes observed in the control arm versus the emulgel arm. Bold values denote changes that are statistically significant (*p* < 0.05).

**Table 3:** Disease severity before and after treatment according to SCORAD and EASI (*n* = 57).

		Before Treatment *n* = 57 (%)	After Treatment *n* = 57 (%)
SCORAD	Mild	37 (64.9%)	46 (80.7%)
	Moderate	14 (24.6%)	9 (15.8%)
	Severe	6 (10.5%)	2 (3.5%)
EASI	Mild	47 (82.5%)	53 (93.0%)
	Moderate	10 (17.5%)	4 (7.0%)
	Severe	0 (0%)	0 (0%)

## Data Availability

The data presented in this study are available on request from the corresponding author. The data are not publicly available due to the privacy of the patients who assisted in the research.

## Abbreviations

AD	Atopic Dermatitis
TEWL	Transepidermal water loss
SCH	Stratum corneum hydration
SCORAD	SCORing Atopic Dermatitis
EASI	Eczema Area and Severity Index
